# Spectroscopic Study of Quantized Breakdown Voltage States of the Quantum Hall Effect

**DOI:** 10.6028/jres.099.068

**Published:** 1994

**Authors:** C. F. Lavine, M. E. Cage, R. E. Elmquist

**Affiliations:** National Institute of Standards and Technology, Gaithersburg, MD 20899-0001

**Keywords:** breakdown of dissipationless state, histograms, quantum Hall effect, quantized vohage states, two-dimensional electron gas, spectra

## Abstract

Quantized breakdown voltage states are observed in a second, wide, high-quality GaAs/AlGaAs sample made from another wafer, demonstrating that quantization of the longitudinal voltage drop along the sample is a general feature of the quantum Hall effect in the breakdown regime. The voltage states are interpreted in a simple energy conservation model as occurring when electrons are excited to higher Landau levels and then return to the original level. A spectroscopic study of these dissipative voltage states reveals how well they are quantized. The statistical variations of the quantized voltages increase linearly with quantum number.

## 1. Introduction

In the integer quantum Hall effect [1] the Hall resistance *R*_H_ of the *i*th plateau of a fully quantized two-dimensional electron gas (2DEG) assumes the values *R*_H_(*i*)*=h/(e^2^i)*, where *h* is the Planck constant, *e* is the elementary charge, and *i* is an integer. The current flow within the 2DEG is nearly dissipationless in the Hall plateau regions of high-quality devices, and the longitudinal voltage drop *V_x_* along the sample is very small. At high currents, however, energy dissipation can suddenly appear in these devices [2,3], and *V_x_* can become quite large. This is the breakdown regime of the quantum Hall effect. The dissipative breakdown voltage *V_x_* can be detected by measuring voltage differences between potential probes placed on either side of the device in the direction of current flow.

Bliek et al. [4] proposed the existence of a new quantum effect to explain the breakdown structures in their curves of *V_x_* versus magnetic field for samples with narrow constrictions. Cage et al. [5] observed distinct quantized *V_x_* states in wide samples. Cage then found that the quantization of these states was a function of magnetic field [6] and current [7]. In this paper we present quantized breakdown voltage data for a second wide sample made from another wafer to give further evidence that there indeed is a new quantum effect. We then investigate how well these breakdown voltages are quantized using a number of experimental techniques.

## 2. Sample

**Fig. 1:**
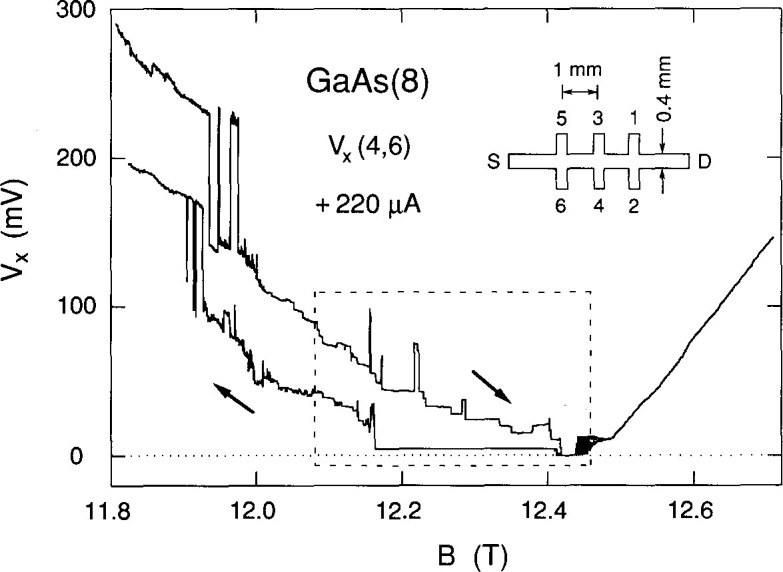
Two sweeps of *V_x_*(4,6) versus *B* for the *i*=2 plateau at +220 µA and 0.33 K. Arrows indicate the sweep directions. The inset displays the sample geometry.

The sample is a GaAs/Al*_x_*Ga_1−_*_x_* As heterostructure grown by molecular beam epitaxy at AT&T Bell Laboratories,^1^ with *x* = 0.29. It is designated as GaAs(8), has a zero magnetic field mobility of 100 000 cm^2^/(V·s) at 4.2 K, and exhibits excellent integral quantum Hall effect properties. This sample and the AT&T GaAs(7) sample used in the previous breakdown experiments [3,5–8] have been used as the United States resistance standard. The inset of Fig. 1 shows the sample geometry. It is 4.6 mm long and 0.4 mm wide. The two outer Hall potential probe pairs are displaced from the central pair by ± 1 mm. The magnetic field is perpendicular to the sample; its direction is such that probes 2, 4, and 6 are near the potential of the source S, which is grounded. Probes 1, 3, and 5 are near the drain potential D. The dissipative voltages *V_x_* for this paper were measured between potential probe pair 4 and 6, hereafter denoted as *V_x_*(4,6) ≡ *V_x_*(4)−*V_x_*(6).

## 3. Longitudinal Voltage Versus Magnetic Field

Figure 1 shows two sweeps of *V_x_*(4,6) versus the magnetic field *B* for the *i* = 2 (12,906.4 Ω) quantized Hall resistance plateau at a temperature of 0.33 K and a current *I* of + 220 µA, where positive current corresponds to electrons entering the source and exiting the drain. This current is approaching the 227 µA critical current value above which, in this magnetic field region, *V_x_* is non-zero for these particular potential probes.

**Fig. 2:**
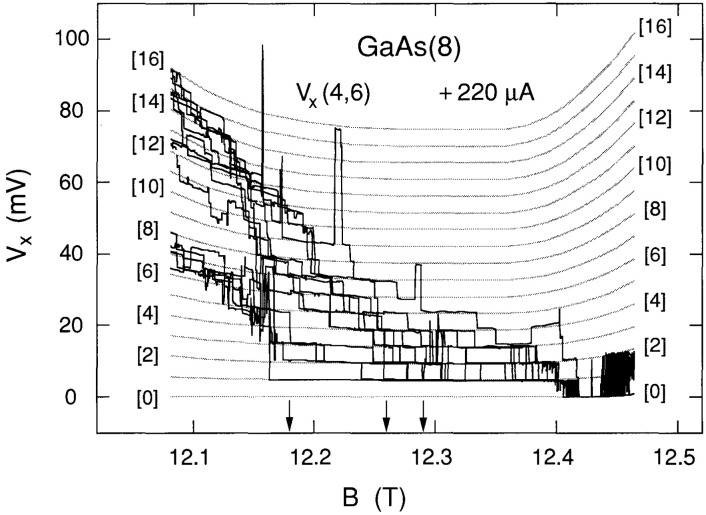
Fourteen sweeps of *V_x_*(4,6) versus *B* at +220 µA, plus a family of 17 shaded curves fitted to these data. The shaded curves were generated with an accuracy of ~1 % and a resolution of ~0.1 %. Voltage quantization numbers are shown in brackets. The vertical arrows indicate magnetic field values of 12.18 T, 12.26 T, and 12.29 T, at which the data shown in Figs. 3–5, and 7–10 were obtained.

Figure 2 shows fourteen sweeps of *V_x_*(4,6) versus *B* over the dashed region of Fig. 1 at the + 220 µA current. The data clearly show discrete, well-defined voltage states, with switching between states. Individual sweeps are not identified in the figure because the magnetic field values at which the states switch have no correlation with sweep number.

We next demonstrate that the discrete voltage states of Fig. 2 are equally separated, and that this separation is a function of magnetic field. This is done by drawing a family of seventeen shaded curves through the data in Fig. 2. The curves have equal voltage separations at each value of magnetic field. The voltage separations are, however, allowed to vary with *B* in order to obtain smooth curves that fit the data. We have argued in Refs. [6,7] that this behavior suggests quantization.

The lowest shaded curve was constrained to be at 0.0 mV everywhere except on the high field side, where a small background voltage was added to provide the best fits as a function of *B;* this deviation from zero voltage presumably arises from some other dissipative mechanism. The 17 shaded curves, which correspond to a *V_x_* = 0.0 mV ground state in the lowest occupied Landau level and 16 excited states, are labeled in brackets as quantum numbers 0 through 16. Deviations of the data from the equally-spaced shaded curves do occur, but the overall trend is clear.

The breakdown activity shown in Fig. 2 is confined to the region between, but not including, the Hall probe pairs 3,4 and 5,6 of Fig. 1. This was demonstrated by measuring the voltages of both Hall probe pairs at this current. The *V*_H_ versus *B* curves of the two Hall probe pairs also had quantized structures, but they occurred over different magnetic field regions than *V_x_.* In addition, the *V_x_* signals were the same on both sides of the sample for probe pairs 3,5 and 4,6.

## 4. Histograms

**Fig. 3:**
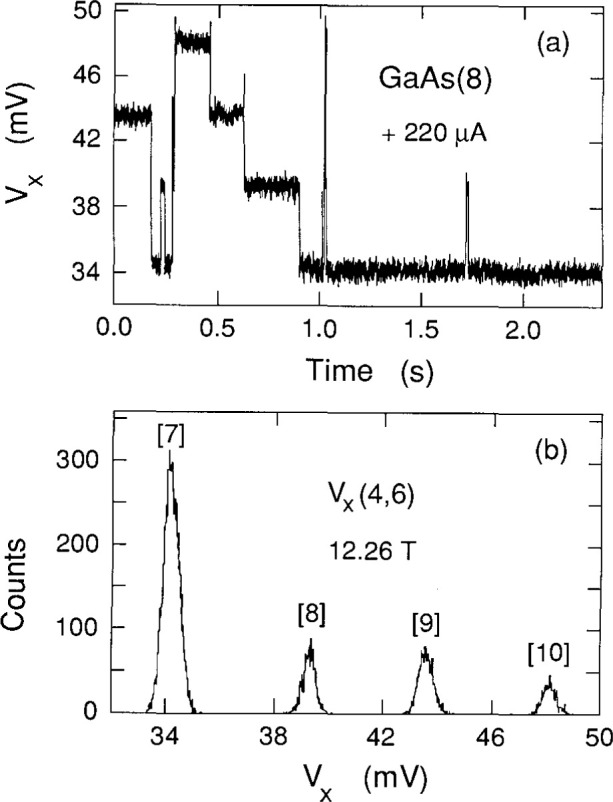
Time sequence of *V_x_* and its histogram at 12.26 T. The numbers in brackets are quantum numbers obtained from Fig. 2.

**Fig. 4:**
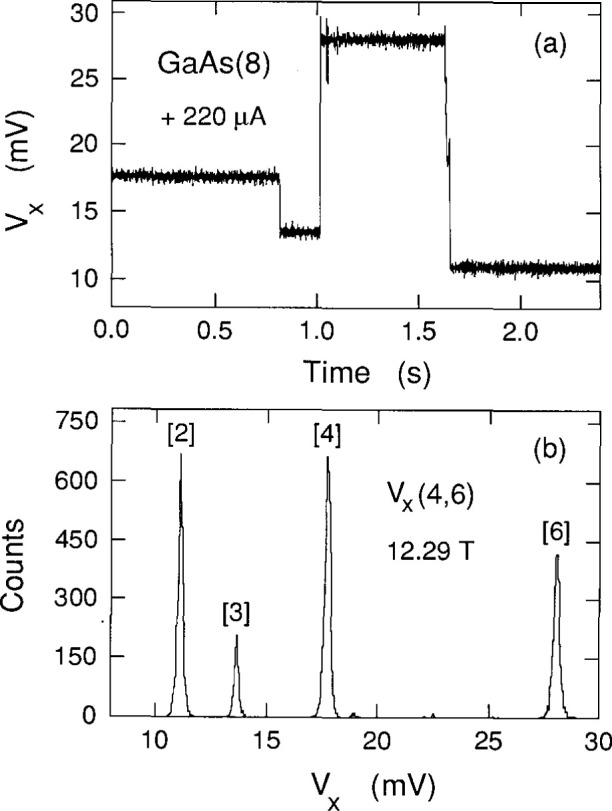
Time sequence of *V_x_* and its histogram at 12.29 T. The numbers in brackets are quantum numbers obtained from Fig. 2.

Cage et al. [8] and Hein et al. [9] have shown that the *V_x_* signal can sometimes be time-averages of two or more discrete dc voltage levels in which only one level is occupied at a time, but where switching occurs between the levels. Therefore, histograms were made to ensure that the signals in Fig. 2 are not time-averages of several levels. Each histogram consists of 16 000 measurements of the *V_x_* signal in a 2.4 s sampling period. They are snapshots in time of the dissipative states and are selected to convey the maximum information. Figure 3(a) shows the time-dependence of one such sampling period at 12.26 T; Fig. 3(b) shows the associated histogram. Figure 4 shows another representative histogram at 12.29 T. No histograms yielded any voltage states other than the ones which appear in the shaded curves of Fig. 2.

**Fig. 5:**
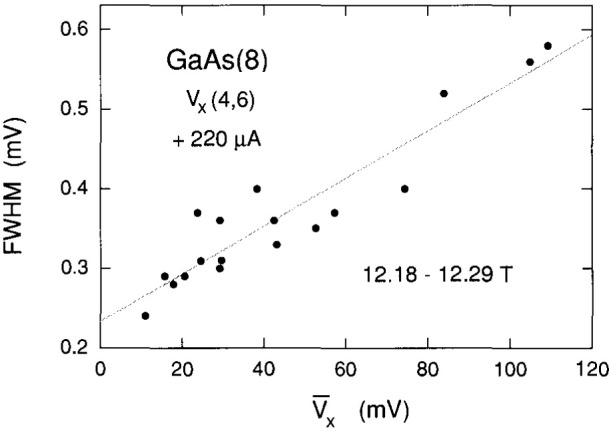
The full-width-at-half-maximums (FWHM) of the histogram peaks that were large enough to obtain adequate measurements. They are plotted versus the histogram peak centroids. The shaded line is a linear least-squares fit to the data.

The histogram peaks are much sharper in Fig. 4 than in Fig. 3, which suggests that the peak widths increase with quantum number. This is investigated in Fig. 5 by plotting the full-width-at-half-maximums (FWHM) of all the prominent histogram peaks observed versus the peak centroids ${\bar{V}}_{x}$. The plot is linear with voltage. If the peak widths are a measure of the lifetimes of the excited states, then the lifetimes decrease with increasing quantum number.

## 5. Simple Model

Many explanations have been proposed [10–17] for the complicated nonlinear breakdown phenomenona. In order to avoid controversy about which explanation is appropriate, we use a simple model [6] based on energy conservation arguments to interpret the voltage quantization displayed in Fig. 2. The breakdown region between the Hall probe pairs 3,4 and 5,6 is treated as a black box. The dissipation is assumed to arise from transitions in which electrons from the originally full Landau levels are excited to states in higher Landau levels and then return to the lower Landau levels. The electrical energy loss per carrier for *M* Landau level transitions is *Mħω*_c_, where *ω*_c_ = eB/m* is the cyclotron angular frequency and m* is the reduced mass of the electron (0.068 times the free electron mass in GaAs). The power loss is *IV_x_.* If (a) the ground state involves several filled Landau levels, (b) only electrons in the highest-filled Landau level make transitions, and (c) electrons of both spin sublevels of a Landau level undergo transitions, then *IV_x_ =r*(2/*i*)*Mħω*_c_, where *r* is the total transition rate and *i* is the Hall plateau number. Thus

$$fM=\left(\frac{re}{I}\right)M=\left(\frac{i}{2}\right)\left(\frac{m^{*}}{\hbar }\right)\left(\frac{V_{x}}{B}\right),$$ (1)

where *f* is the ratio of the transition rate *r* within the breakdown region to the rate *I/e* that electrons transit the device; *f* can also be interpreted as the fraction of conducting electrons that undergo transitions.

We associate the quantized values of *M* with the numbers in brackets for the shaded curves in Fig. 2. *I*, *V_x_*, and *B* are measured quantities, and i, m*, and *ħ* are constants. Therefore, *f* and *r* can be determined from the *V_x_* versus *B* plots and Eq. (1) because *M* is known.

**Fig. 6:**
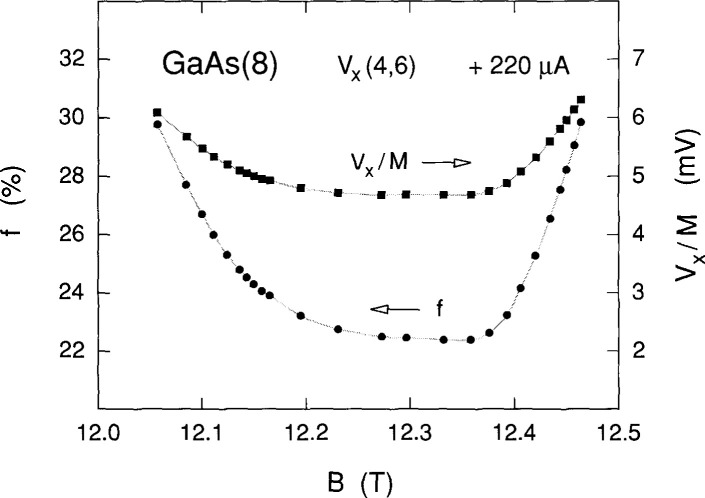
The voltage quantization *V_x_*/*M* and the fractions *f* (expressed as a percentage) of electrons making the Landau level transitions for the seventeen shaded curves shown in Fig. 2 at + 220 µA. See Eq. (1) for the definition of *f*.

Figure 6 shows the variation of the voltage quantization *V_x_/M* over the magnetic field range of Fig. 2. This quantization is model-independent, except for assigning the quantum numbers *M* to the shaded curves. *V_x_/M* varies within the range 4.68 mV to 6.30 mV. The fractions *f* (expressed as a percentage) of electrons that make the transitions in the shaded curves of Fig. 2 were calculated using Eq. (1), and are also shown in Fig. 6; *f* varies between 22.4 % and 29.8 %, corresponding to transition rates between 3.1 × 10^14^/s and 4.1 × 10^14^/s. The large numbers of electrons involved in these transitions imply a collective effect.

## 6. Spectra

The voltage states are clearly quantized, but how *well* are they quantized? Voltage spectra would be useful to address this question. Histograms are not themselves spectra because the areas under the peaks do not correspond to the excitation probabilities. Many histograms must be accumulated to obtain a spectrum. This is very time-consuming. Therefore, we devised another method to obtain voltage spectra by momentarily pushing the sample current to 390 µA at a fixed magnetic field and then reducing it back to 220 µA. This procedure induced the dissipative dc voltage states that were then recorded.

**Fig. 7:**
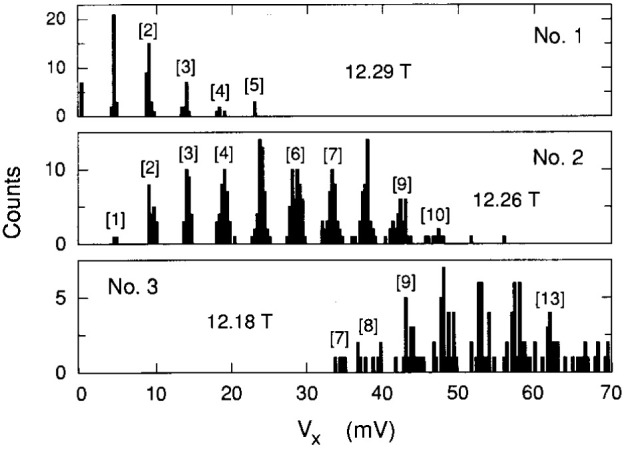
Three voltage spectra taken at the *B* values indicated by the arrows in Fig. 2. The numbers in brackets are the *M* values.

**Fig. 8:**
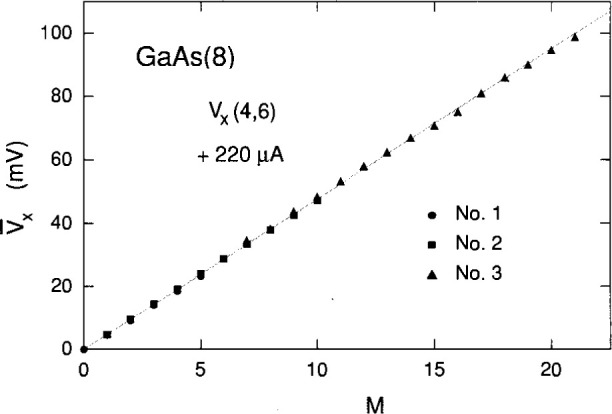
Average value ${\bar{V}}_{x}$ of each voltage peak of the three spectra in Fig. 7 versus the quantum number *M*, plus a shaded linear least-squares fit to the data.

Three voltage spectra are shown in Fig. 7. Spectra 1 and 2 correspond closely to the *V_x_* versus *B* sweeps in Fig. 2, but the pulsed current induced much higher states in spectrum 3 then observed in Fig. 2. This is due to a bifurcation effect in which a second range of states can be excited, as was observed in GaAs(7) [6,7]. Figure 8 plots the ${\bar{V}}_{x}$ of each peak of the three spectra in Fig. 7 versus the quantum number *M.* The shaded line is a least-squares fit to the data. The fit provides an average value of the dissipation voltage per quantum level, ${\bar{V}}_{x}/M$, of 4.76 mV, and a corresponding average *f* value of 22.9 %.

**Fig. 9:**
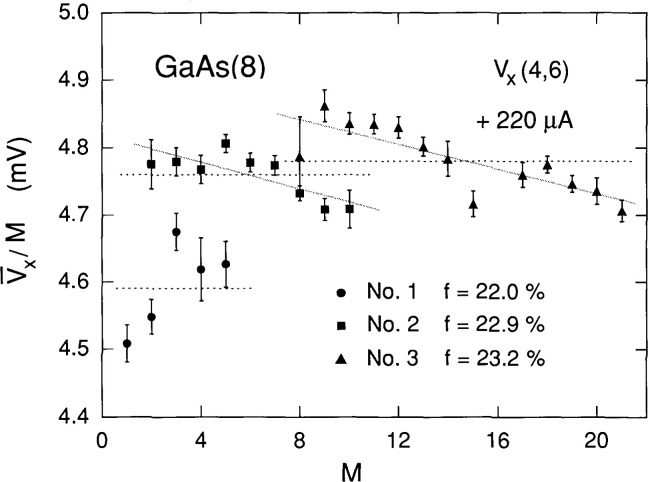
Mean values of ${\bar{V}}_{x}/M$ for the three spectra in Fig. 7 plotted versus *M.* Only peaks with at least seven counts are included for spectra 2 and 3. The error bars represent one-standard-deviation uncertainties. There is more scatter in the values for spectrum 1 because ${\bar{V}}_{x}$ is divided by smaller values of *M.* Horizontal dashed lines are weighted averages of ${\bar{V}}_{x}/M$; corresponding *f* values are also displayed. The two shaded lines are least-squares fits that were weighted by the measurement uncertainties.

The linear fit in Fig. 8 is excellent, but we know from the family of shaded curves in Fig. 2 that the values of ${\bar{V}}_{x}/M$ and *f* vary with *B*. Therefore, ${\bar{V}}_{x}/M$ is plotted versus *M* in Fig. 9 for the three spectra in Fig. 7. The *f* values, corresponding to the horizontal dashed lines representing the weighted means of ${\bar{V}}_{x}/M$, are within 0.5 % of those obtained from the shaded curves of Fig. 2. The two shaded lines in Fig. 9 are weighed least-squares fits to spectra 2 and 3; they suggest a tendency for the voltage quantization to decrease with increasing *M* values at a constant *B.* This decrease provides a cautionary note about the degree of quantization, and also about the assumptions in the simple black box model that the values of *f* and *r* remain constant for increasing *M* at constant *B.* However, this apparent quantization decrease with increasing *M* is a small effect, and it does not seriously affect interpretation of the data–as evidenced by the fit in figure 8.

**Fig. 10:**
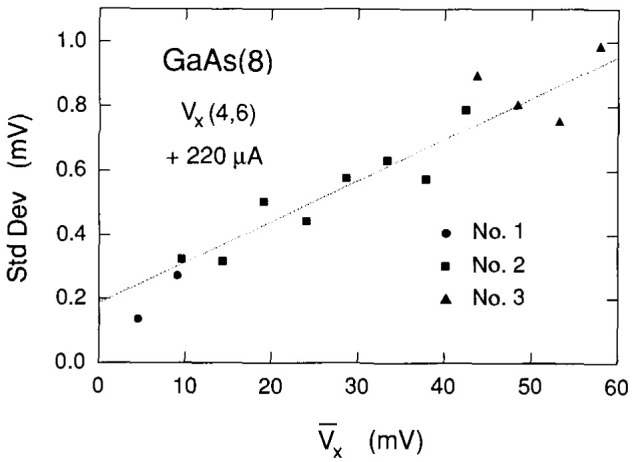
Standard deviations of those peaks of the spectra in Fig. 7 that contain at least eighteen counts plotted versus the peak centroids, plus a linear least-squares fit to the data.

Another measure of the degree of quantization is the sharpness of the spectra peaks. This is explored in Fig. 10. The standard deviations of those peaks of the spectra in Fig. 7 that contain at least eighteen counts are plotted versus the peak centroids. There is a linear increase in peak width with quantum number, perhaps due to a decrease in lifetimes for higher-lying excited states, just as there was for the histograms. The statistical fluctuations of the voltage quantization increase linearly with increasing quantum number.

## 7. Conclusions

Quantized dissipative voltage states clearly exist in the breakdown regime of the quantum Hall effect. This quantization is interpreted in a simple model as occurring when electrons make transitions from a lower Landau level to a higher level and then return to the lower level. The large *V_x_* signals imply a high transition rate and a collective effect. Voltage quantization suggests that individual electrons either make a single transition, or a fixed number of multiple transitions, because varying numbers of transitions would result in a continuum of *V_x_* values rather than voltage quantization.

The data presented here are very striking, with sharp vertical features in *V_x_* versus *B* plots, switching between states, and sufficient variations between sweeps to generate families of shaded curves, detailed histograms, and sharp spectra, and thereby to unambiguously determine values of the quantum number *M.* The voltage quantization is not perfect. It may decrease slightly with increasing quantum number, and its statistical variation increases linearly with quantum number. Still, the degree of quantization is quite surprising.

## Footnotes

1 Certain commercial equipment, instruments, or materials are identified in this paper to foster understanding. Such identification does not imply recommendation or endorsement by the National Institute of Standards and Technology, nor does it imply that the materials or equipment identified are necessarily the best available for the purpose.
